# Frequent Infection of Human Cancer Xenografts with Murine Endogenous Retroviruses *in Vivo*

**DOI:** 10.3390/v7042014

**Published:** 2015-04-17

**Authors:** Asif Naseer, Anne Terry, Kathryn Gilroy, Anna Kilbey, Ciorsdaidh Watts, Nancy Mackay, Margaret Bell, Susan Mason, Karen Blyth, Ewan Cameron, James C. Neil

**Affiliations:** 1 Molecular Oncology Laboratory, MRC-University of Glasgow Centre for Virus Research, University of Glasgow, Garscube Estate, Bearsden Road, G61 1QH Glasgow, UK; E-Mails: a.naseer.1@research.gla.ac.uk (A.N.); anne.terry@glasgow.ac.uk (A.T.); kathryn.gilroy@glasgow.ac.uk (K.G.); anna.kilbey@glasgow.ac.uk (A.K.); ciorsdaidh.watts@glasgow.ac.uk (C.W.); annie.mackay@glasgow.ac.uk (N.M.); 2 School of Veterinary Medicine, University of Glasgow, Garscube Estate, Bearsden Road, G61 1QH Glasgow, UK; E-Mails: margaret.bell@glasgow.ac.uk (M.B.); ewan.cameron@glasgow.ac.uk (E.C.); 3 Cancer Research UK Beatson Institute, Garscube Estate, Switchback Road, Bearsden, G61 1BD Glasgow, UK; E-Mails: s.mason@beatson.gla.ac.uk (S.M.); k.blyth@beatson.gla.ac.uk (K.B.)

**Keywords:** xenograft, xenotropic, murine leukemia virus, cancer

## Abstract

Infection of human cancer xenografts in mice with murine leukemia viruses (MLVs) is a long-standing observation, but the likelihood of infection *in vivo* and its biological consequences are poorly understood. We therefore conducted a prospective study in commonly used xenograft recipient strains. From BALB/c nude mice engrafted with MCF7 human mammary carcinoma cells, we isolated a virus that was virtually identical to *Bxv1*, a locus encoding replication-competent xenotropic MLV (XMLV). XMLV was detected in 9/17 (53%) independently isolated explants. XMLV was not found in primary leukemias or in THP1 leukemia cells grown in *Bxv1*-negative NSG (NOD/SCID/γCnull) mice, although MCF7 explants harbored replication-defective MLV proviruses. To assess the significance of infection for xenograft behavior *in vivo*, we examined changes in growth and global transcription in MCF7 and the highly susceptible Raji Burkitt lymphoma cell line chronically infected with XMLV. Raji cells showed a stronger transcriptional response that included up-regulation of chemokines and effectors of innate antiviral immunity. In conclusion, the risk of *de novo* XMLV infection of xenografts is high in *Bxv1* positive mice, while infection can have positive or negative effects on xenograft growth potential with significant consequences for interpretation of many xenograft studies.

## 1. Introduction

The potential for acquisition of endogenous murine retrovirus infection by human cells grown as xenografts was first reported many years ago, prior to the advent of molecular cloning and sequence-based classification of these viruses [1,2]. However, this phenomenon and its relevance to research on human disease were largely ignored until reports of XMRV (xenotropic murine leukemia virus-related virus) as a potential human pathogen brought these issues back into the limelight. While XMRV has been discounted as a cause of human disease and the misleading evidence accounted for by contamination of clinical material and testing kits, forensic investigation traced the origins of this virus to the xenograft-derived human prostate cancer cell line 22Rv1 that had been passaged in mice [3]. No precise endogenous counterpart of XMRV was identified as this virus appears to be a recombinant between two endogenous xenotropic murine leukemia viruses (XMLVs), designated PreXMRV-1 and PreXMRV-2 [4].

Adventitious infection of well-established cell lines with gamma-retroviruses is not a new phenomenon [5] and recent large scale sequence analyses of human cancer cell lines have identified the presence of murine leukemia viruses in a number of widely used lines, including a subset of Burkitt lymphoma cell lines [6,7]. However, the difficulty in tracing the source and assessing the wider significance of these reports was illustrated by a study which showed that 23% of human cancer cell lines that had been passaged through mice showed evidence of infection with XMLVs while 17% of non-xenograft derived cell lines from the same facility were also infected. Notably, cells maintained in a xenograft-free laboratory were uniformly negative [8]. We therefore decided to conduct a prospective study of XMLV generation in mouse strains frequently used for xenograft studies. Our results show that there is a very significant risk of acquiring XMLV in human breast cancer-derived MCF7 xenografts grown in BALB/c nude mice, a commonly used recipient strain.

The hazards arising from XMLV infection are of potential interest to scientists from many fields where xenografting is used as a routine research tool [9,10,11], and this has been the subject of a recent review [12]. The extent of xenograft use is evident from a keyword search in PubMed, which at the time of writing revealed almost 32,000 entries. Furthermore, recent reports suggest that XMLVs are not merely an unwanted passenger with biohazard potential as they are likely to influence the behavior of xenografts *in vivo* [13,14]. Our initial analysis of the transcriptomes of two human cell lines infected with XMLV suggests that infection may elicit an innate immune response that favors xenograft rejection as well as altered cell behavior relevant to cancer phenotype analysis.

## 2. Materials and Methods

### 2.1. Cell Lines and Virus Infections

Cells were grown in MEM (10370047 Gibco, Life Technologies, Paisley, UK) (MCF7) RPMI 1640 (21875 Gibco: DERSE, CEM, CEMSS, Kyo1, K562, U937, Jurkat, Raji, Reh, PBMC, THP-1), DMEM (31966 Gibco: 293T, MDA MB 231), DMEM (31885 Gibco: AH927) or Stem Span medium (Stem cell technologies: CD 34+ human cord blood stem cells). All growth media were supplemented with 10% FCS (HyClone, Perbio, Life Technologies, Paisley, UK), 100 μg/mL Penicillin/Streptomycin (15140 Gibco), and 2mM Glutamine (25030 Gibco). Additional media supplements were added as follows: 0.01 mg/mL Insulin (10 mg/mL, I9278 Sigma, Munich, Germany) and 1 mM Sodium pyruvate (11360 Gibco) were added to MCF7 cells; 1 μg/mL Puromycin (P9620 Sigma) was added to DERSE cultures, 0.05 mM 2-mercaptoethanol (M7522 Sigma) was added to Reh and THP-1 cells. CD34+ cells were supplemented with FGF-1 10 ng/mL (Miltenyi Biotec, Surrey, UK), SCF 20 ng/mL (Miltenyi Biotec), TPO 10 ng/mL (Miltenyi Biotec), IGF BP2 100 ng/mL (Miltenyi Biotec), ANGPTL5 500 ng/mL (Miltenyi Biotec), Heparin 10 μg/mL (Sigma). PBMCs were grown in the presence of 5 μg/mL PHA (Sigma) and 200 units/mL human IL2 (Peprotech, London, UK). PHA stimulation of PBMCs was limited to the initial 3 days of culture. All cell cultures were maintained at 37 °C in 5% CO_2_.

Cells were infected with FeLV-B from sub-confluent infected AH927 feline fibroblasts or XMLV from infected MCF7 cells. Cells were exposed to 0.45 µm filtered supernatants. Medium was replaced after 2 h of infection and cells were cultured for 15 days. DNA extraction was carried out using a DNeasy kit (Qiagen, Manchester, UK). DERSE cells were kindly provided by Dr. V. KewalRamani (National Cancer Institute, Frederick, MD, USA).

### 2.2. Southern Blot Hybridization of Single Cell Clones

Single cell cloning was achieved by serial 1 in 2 dilution of a cell suspension of MCF7 XMLV into a 96 well plate containing 50% MCF7 conditioned medium in normal medium. Wells with single cells were marked next day and grown for 10 days with replacement of conditioned medium every 3–4 days. DNA was extracted using the DNeasy kit, digested with EcoRV, Southern blotted and hybridised as previously described [15] with a 380 bp probe specific to the LTR region of XMLV generated by pcr amplification (primers XMLV probe F 5' GATGGTACTCAGATAAAGCGAAACT 3' and XMLV probe R 5' CTGGGTAGTCAATCACTCTGAGG 3').

### 2.3. RNA Extraction and Microarray Analysis

Total RNA was isolated by RNeasy kit (Qiagen, Manchester, UK) from three cultures each of uninfected and XMLV infected MCF7 and uninfected and XMLV infected Raji cells. RNA was tested for quality on the Agilent 2100 Bioanalyser (Agilent Technologies, Stockport, UK) and NanoDrop 2000 (Thermo Scientific, Walatham, MA. USA) before screening against Affymetrix GeneChip Human Gene 2.0 ST array (High Wycombe, UK, 2014) by ATLAS Biolabs (Berlin, Germany).

### 2.4. Quantitative Real-Time PCR

cDNA synthesis was performed using the QuantiTect Reverse Transcription Kit (Qiagen, Manchester, UK) as previously described [16]. 12.5 ng aliquots of cDNA were amplified in triplicate on ABI 7500 Sequence Detection System using Power SYBR Green PCR Master Mix (Life Technologies, Paisley, UK) and QuantiTect primers for human endogenous control HPRT (QT00059066) or IFI44 (QT00014399), CCNB1 (QT00006615), PCOLCE2 (QT00003479) TSPAN2 (QT00046480), CCL4 (QT01008070), IL7R (QT00053634), SLC30A4 (QT00031255) to validate gene expression changes.

### 2.5. Wound Healing Assay

MCF7 cells (XMLV infected and non-infected) were seeded in a 6 well plate and grown to confluence. Wounds were generated with a pipette tip, washed with PBS and fresh medium added. Areas with identical wound sizes were then followed for 3 days with serial image capture using a Leica camera. Wound size was calculated using Image J and graphs plotted using Microsoft Excel.

### 2.6. PCR Detection of Bxv-1 and XMLV

100 ng aliquots of mouse genomic DNA were amplified using primers for either *Bxv1* (*Bxv1*- F 5' GGCCTCGCTGTTCCTTG 3' and *Bxv1*-R 5' GAGAGAGCGTGGCAAACCTT 3') or murine GAPDH (mmGAPDH-F 5' AGTATGATGACATCAAGAAGG 3' and mmGAPDH-R 5' ATGGTATTCAAGAGAGTAGGG 3') in 2× Reddy Mix (Thermo Scientific, Manchester, UK) at 95 °C 3 min then 35 cycles 95 °C 30 s, 60 °C 30 s, 72 °C 3 min. PCR products were separated on a 2% agarose TAE gel. Primers to detect XMLV LTR (Fn8 5' CTGGATCTATTGATTTGAGTTGG 3' and Rn8 5' GCTTTATTGGGAACACGGGTA 3') and Human GAPDH (Hs GAPDH F 5' CCCCACACACATGCACTTACC 3' and Hs GAPDH R 5' CCTAGTCCCAGGGCTTTGATT 3').

### 2.7. Animal Experiments

MCF7 cells (5 × 10^6^ cells) suspended in 50 μL Matrigel were injected subcutaneously into BALB/c nude mice (Charles River) with mice receiving oral oestrogen (Sigma Aldrich) in the drinking water (1 ug/mL) for at least a week before the inoculations were performed. In subsequent experiments MCF7 cells (5 × 10^6^ cells) suspended in 50 μL Matrigel were inoculated into BALB/c nude and NSG mice (NOD.Cg-Prkdcscid Il2rgtm1Wjl/SzJ) mice; Jackson Laboratories, Bar Harbor, ME, USA) by intramammary xenograft under general anaesthesia. In these experiments a slow release oestrogen pellet (Innovative Research of America) was implanted subcutaneously into each mouse 24 h prior to MCF7 xenografting. BALB/c nude mice were also transplanted with Raji cell using the subcutaneous route (1 × 10^6^ in 100 µL of PBS) and THP-1 cells (1 × 10^6^ in 100 µL of PBS) using the intravenous (tail vein) route. NSG mice were conditioned using a sub-lethal dose of 1Gy whole body radiation and transplanted with THP cells (1 × 10^6^ in 100 µL of PBS) by intravenous (tail vein) injection 24 h later. All animals were humanly culled at experimental end points in full compliance with UKCCCR (United Kingdom Coordinating Committee on Cancer Research) protocols.

Mice were housed in individual ventilated cages, supplemented with trimethoprim and sulfadiazine in the drinking water (Tribrisson, Schering-Plough Animal Health, final concentration 0.048%) and all manipulations performed in a laminar flow hood. Animal protocols used in this work were evaluated and approved by the University of Glasgow Ethics and Welfare Committee and were carried out under Home Office License (approval granted September 2012, license number PPL 60/4408) as governed by the Animal Scientific Procedures Act, 1986.

### 2.8. Phylogenetic Analyses

*De novo* rescued viral sequences were searched for similarity to entries in the NCBI database using the Basic Local Alignment Search Tool (http://blast.ncbi.nlm.nih.gov/). Trees were generated by the neighbour-joining method with bootstrapping using CLC Genomics Workbench software.

### 2.9. Microarray Analysis

Independent cultures of both MCF7 and Raji cell lines were revived and grown in parallel in identical conditions for three passages. The virus producing MCF7 cells were plated at 2 × 10^5^ cells/mL. After 48 h, the supernatant was used to infect 1 × 10^6^ MCF7 and Raji cells in triplicates. This gave three biological replicates of infected and uninfected cells per cell line. Cells were grown for two weeks before harvesting. Suspension Raji cells, were washed twice with PBS. MCF7 cells were washed twice in PBS, trypsinised, washed twice more in PBS to make cell pellets. RNA was extracted from the cells using RNeasy kit (Qiagen) with DNAse digestion as per manufacturers protocol, then quality assessed with Agilent 2100 Bioanalyser (RIN 9 and over accepted) and Nanodrop spectrophotometer (Thermo Scientific, Walatham, MA, USA) (260:280 > 1.5 and 260:230 ratio > 1 accepted).

Expression analysis (RNA labelling, hybridization, staining, scanning and background correction) was carried out on Affymetrix HuGene 2.0ST chips (High Wycombe, UK) by the commercial provider Atlas Biolabs according to standard protocols. Affymetrix Expression Console software with RMA normalization (High Wycombe, UK, 2014)was used to generate.chp files, and then CLC Workbench Genomics Workbench software was used to perform statistical analysis, including fold-changes, *p*-value statistical significance, and multiple testing correction (Bonferroni or FDR). Raw intensity.cel files, processed.chp files and data summary spreadsheets are available for download.

## 3. Results

### 3.1. Frequent Recovery of XMLV from MCF7 Xenografts in BALB/c Mice

**Figure 1:**
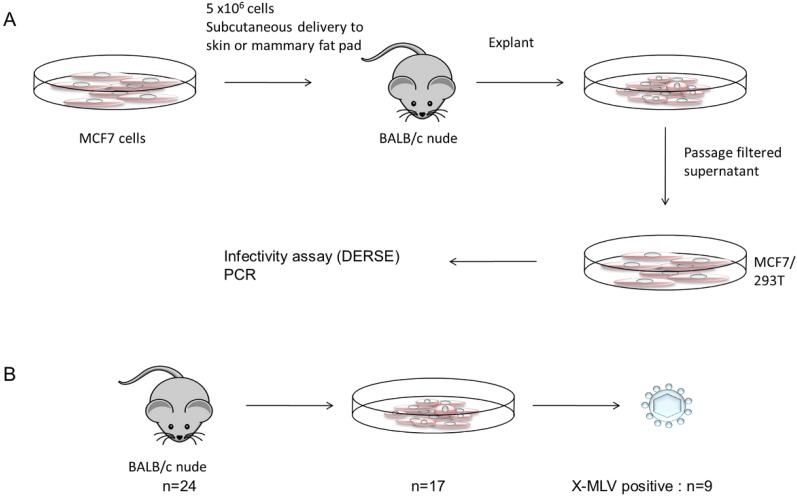
Experimental design and key findings from xenografting of MCF7 cells in BALB/c mice. In an initial experiment (**A**) cells were inoculated subcutaneously and thereafter by the intra-mammary route. Growing tumors were explanted and culture supernatant was filtered to remove contaminating cells before infection of fresh MCF7 or 293T cells. After passage to allow virus spread (2 weeks) cultures were assayed for the presence of replicating virus using the DERSE assay [21] with confirmation by virus-specific PCR. This process was repeated in further experiments involving intra-mammary inoculation. (**B**) summarises the cumulative results. In total, 24 xenografts gave rise to 17 growing tumors and viable explants, of which nine were positive for replicating XMLV.

To assess the likelihood of acquiring *de novo* infection with murine leukemia virus we used the MCF7 mammary carcinoma cell line [17], a well-established xenograft model which we had previously found to be highly susceptible to infection with another gamma-retrovirus, feline leukemia virus (FeLV). BALB/c mice were chosen as recipients in light of previous evidence of inducible xenotropic virus in this strain [18,19,20]. In a pilot experiment four female BALB/c nude mice were engrafted subcutaneously with 5 × 10^6^ MCF7 cells. One mouse developed a growing tumor that was harvested and explanted *in vitro*. The presence in the explant cells of retroviruses with the ability to enter human cells was initially tested and demonstrated using the DERSE assay (Detector of Exogenous Retroviral Sequence Elements) [21] as shown in Figure 1. This assay makes use of a retroviral construct containing an intron-interrupted GFP reporter gene that is active only after mobilization and transfer to bystander cells. The cell line is based on human prostate cancer cell line LNCaP. The assay detects a range of gammaretroviruses with functionally related packaging sequences. Culture supernatants from the positive explant cells were filtered and passaged onto fresh MCF7 or 293T cells to ensure removal of contaminating mouse cells.

Screening of the passaged virus with specific PCR primers revealed the presence of an XMLV related virus that was detected with several specific primers. Complete sequence analysis after PCR amplification of genome fragment showed that the newly recovered XMLV was virtually identical (99.5%) to the *Bxv1* locus of BALB/c mice [22,23] (Figure S1). Tests with primers specific for BALB/c-derived ecotropic virus [24] were also negative (not shown). It should be noted that our laboratory had not previously worked with XMLV and that the stock MCF7 cells used for inoculation have at no time tested positive for XMLV. To improve tumor growth, subsequent experiments were undertaken using the intra-mammary route. The explants and cultures were handled separately and fastidiously to ensure that no cross-contamination occurred. In total, 24 mice were inoculated, yielding 17 explanted tumors of which nine were found to be positive for replicating XMLV (Figure 1; panel B). To test other potential substrates in this system we inoculated 8 BALB/c nude mice subcutaneously with Raji Burkitt lymphoma cells (10^6^ cells) but these cells formed only regressing nodules and did not yield viable explants. We also inoculated four BALB/c mice intravenously with THP-1 leukemia cells (10^6^) but these mice remained healthy and showed no detectable human cell engraftment.

**Figure 2:**
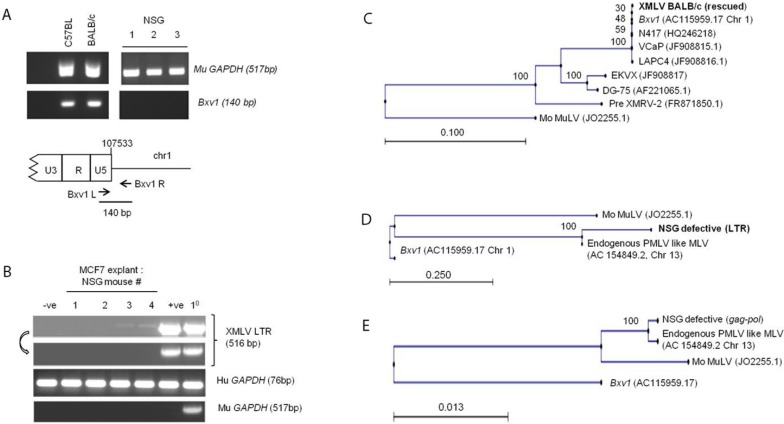
Analysis of endogenous MLVs in NSG mice and recovered viral sequences from xenografts. (**A**) DNA PCR analysis of the *Bxv1* locus in BALB/c, C57BL/6 and NSG mice. Although the breeding history of NSG mice includes the C57/BL6 strain, NSG mice do not contain an XMLV provirus at the *Bxv1* locus. Murine GAPDH gene primers provide the loading control and _ denotes no added DNA control; (**B**) Two out of four MCF7 xenograft explants from NSG mice were DNA PCR positive for MLV primers after one passage of filtered supernatant on fresh MCF7 cells, but this signal was lost after a further round of passage (denoted by arrow). 1° denotes a primary explant which contained significant residual murine cellular DNA sequences. The positive control (+ve) was MCF7 cells infected with passaged XMLV. The negative control (−ve) was uninfected MCF7 cells; (**C**–**E**) Sequences were aligned and phylogenetic trees were generated using the Neighbor Joining method. Scale bars denote genetic distance and bootstrapping values are indicated at the nodes. Bootstrapping values were based on 100 replicates. The tree in c is based on a ~7.5 kb fragment of the newly recovered XMLV from MCF7 xenografts in BALB/c mice. This sequence was virtually identical to *Bxv1* and several independent xenograft-derived isolates. Trees d and e show alignment of LTR and *gag-pol* fragments from the defective virus in MCF7 explants derived from NSG mice as shown in panel b. These sequences align most closely with an endogenous polytropic MLV-like provirus on mouse chromosome 13 (see text).

Finally we also tested MCF7 explants from NSG (NOD-SCID-γC null) mice. This strain is in increasing use for xenografting due to a profound immune deficit and high permissiveness for engraftment with non-murine cells [25]. Although the pedigree of NSG mice includes the C57BL/6 strain which harbors the *Bxv1* locus, PCR analysis showed that NSG mice do not (Figure 2A). Testing of MCF7 mammary fat pad explants (*n* = 4) yielded no replication-competent XMLV. In addition we tested explants from liver tumors induced by THP1 leukemia cells and a number of primary childhood leukemia cell lines (*n* = 4) that had previously been passaged in NSG mice [26], again without any positive finding for XMLV infection.

While no replication-competent viruses emerged, initial passage of NSG explant supernatants on MCF7 cells showed evidence of positive PCR signals with XMLV primers (Figure 2B). Cloning and sequence analysis revealed LTR and *gag-pol* sequences from a population of proviral sequences readily distinguished from *Bxv1* but clearly related to other endogenous murine retroviral elements (Figure 2C, Figure S1). The NSG-derived virus was close to identical to database entries for endogenous polytropic MLV proviruses derived from a C57BL/6J BAC library. Differences were limited to several G to A transitions, suggesting cytidine deamination during replication as a possible source of divergence [27].

### 3.2. Susceptibility of Human Cell Lines to Recovered XMLV Infection

**Figure 3:**
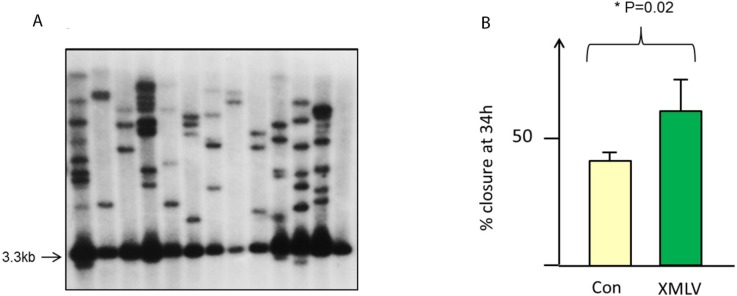
High copy number and phenotypic effects of XMLV in MCF7 cells. (**A**) Depicts Southern blot analysis of single cell clones of XMLV-infected MCF7 cells. DNA was digested with EcoRV and probed with an LTR-*gag* probe from XMLV. This analysis detects an internal genomic fragment of 3.3.kb and a series of unique integration host-virus junction fragments; (**B**) Accelerated wound healing in chronically infected MCF7 cells compared to control uninfected cells (significance was determined by unpaired t test).

We noted that XMLV was readily detected in freshly infected MCF7 cells. Many established human cell lines were found to be susceptible to XMLV infection although most did not accumulate copy numbers as high as that observed in MCF7 cells and primary PBMCs were found to be refractory to productive infection as previously noted for XMRV [28] (Figure 2B; Table 1). While DERSE cells were useful for detection of high titre virus, we found this cell system to be relatively insensitive as a method of virus titration, which was therefore carried out by a quantal dilution assay on MCF7 cells, indicating a maximal titre of 10^4^ infectious units/mL with lower titre viruses. In light of this relatively low titre, we were surprised to discover XMLV proviral copy numbers ranging from 2 to 15 per cell by Southern blot analysis of single cell clones from infected MCF7 cultures. The blot analysis revealed numerous single copy and unique host-virus EcoRV junction fragments in addition to the common internal proviral fragment of 3.3 kb (Figure 3a).

**Table 1:** Susceptibility of human cells and cell lines to infection with *in vivo* recovered XMLV.

Target cell	Origin	Susceptibility ^a^	Titre ^b^
MCF7	Mammary carcinoma	++++	>10^4^
CEM	T-cell leukemia (ALL)	+++	10^1^
CEM-SS	CEM subclone	+++	10^1^
K562	Myelogenous leukemia (CML)	+++	ND
293T	Embryonic kidney	++++	>10^2^
Raji	Burkitt lymphoma	+++++	ND
CD34+	Cord blood	++	ND
Kyo-1	Myelogenous leukemia (CML)	++	0
U937	Histiocytic lymphoma	++	ND
Jurkat	T-cell leukemia	++	ND
THP-1	Monocytic leukemia	++	ND
MDA MB 231	Mammary carcinoma	++	ND
Reh	Acute lymphocytic leukemia	+	0
PBMC	Primary blood mononuclear	+	0

^a^ Based on quantitative PCR analysis of proviral DNA; ^b^ Based on dilution assay on MCF7 cells and endpoint determination by DNA PCR for XMLV.

### 3.3. Phenotypic Effects of XMLV Infection

There have been a number of reports of altered cell behavior after XMLV or XMRV infection of human cells [13,14,28]. While we observed no gross effect of infection in MCF7 we carried out a number of tests for subtle alterations in behavior. In particular, we noted a small but significant (*p* = 0.02) increase in the rate of wound healing in XMLV infected cells compared to uninfected controls (Figure 3B). A similar effect was noted in FeLV-B infected MCF7 cells, indicating a general retroviral effect rather than the result of specific receptor ligation [13]. While this effect was highly reproducible it was observed only in cells with established infection (>14 days) and was not observed in newly infected cells. In contrast to a published report [28] we did not observe any cytopathic effect induced by XMLV in the Raji Burkitt lymphoma cell line, despite the accumulation of copy numbers similar to those observed in MCF7 cells.

### 3.4. Effects of XMLV Infection on the Human Transcriptome

**Figure 4:**
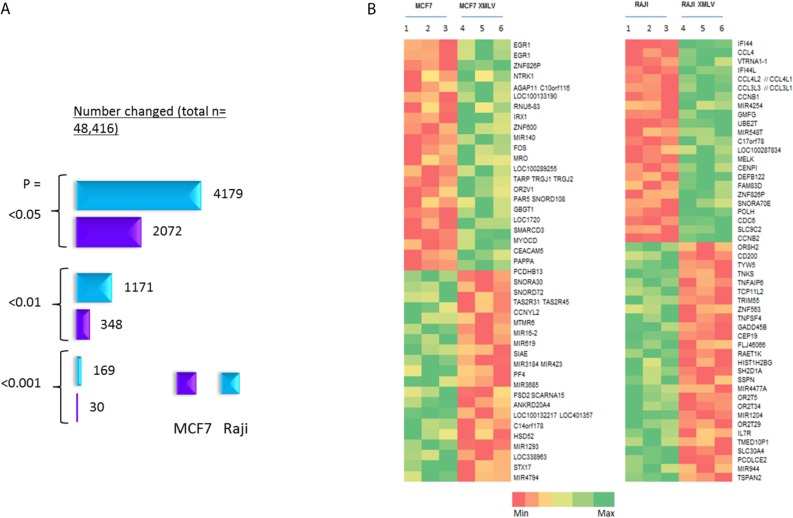
Gene expression microarray analysis (Affymetrix HuGene 2.0ST) of XMLV infected MCF7 and Raji cells. (**A**) Number of changed probe sets at different *P* value cut-offs; (**B**) Heat map of the most highly up- and down-regulated genes in XMLV-infected MCF and Raji cells, based on a *p* = <0.05 and fold change >1.5 cut-off.

To assess the effects of XMLV infection at the transcriptome level we carried out gene expression microarray analyses of MCF7 and Raji cells. Three biological replicates were included for uninfected and infected cultures, and effects on global gene expression were analysed using Affymetrix Human Gene 2.0ST chips. While many changes were detected, the fold-changes involved were generally low and few passed standard false discovery thresholds due to the large number of probes analysed on each chip. Nevertheless, validation confirmed changes indicating real differences resulting from XMLV infection. Moreover, there was a striking difference between Raji and MCF7 cells in the magnitude of the response, with the former displaying almost ten times as many probe sets passing a threshold defined by *P* = <0.05 and fold change >1.5 (Figure 4). It was also notable that the transcriptional changes in MCF7 showed a higher percentage of non-coding RNAs (Supplementary Data F1).

Heat maps of the most substantially changed probe sets in each cell line are shown in Figure 4. There was no overlap in the gene sets, implying that distinct biological processes may be triggered by infection in each cell type. In the case of MCF7 cells the gene displaying the largest fold change was for *EGR1*, a growth regulatory gene which was up-regulated in XMLV infected cells as indicated by two separate probe sets, and validated by quantitative (qt) RT-PCR as a 2-fold change.

**Figure 5:**
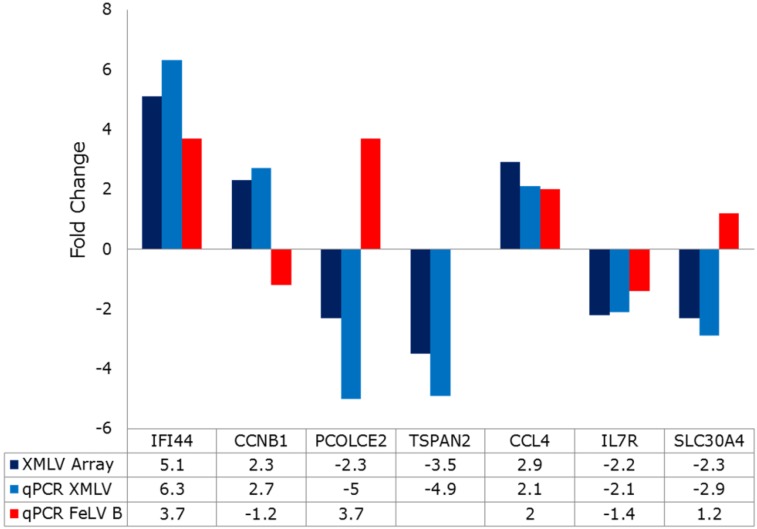
Validation of significant gene expression changes in XMLV-infected Raji cells by quantitative RT-PCR. Fold changes measured by the microarray shown in Figure 4 (dark blue bars) were validated by qtRT-PCR (light blue bars) and the same genes compared in FeLV-B infected Raji cells (red bars). Fold changes are also listed in the table below. Note that all microarray changes were validated, while only a subset of genes are similarly regulated in FeLV-B-infected Raji cells. NA: not analysed.

The most prominent changes seen in Raji cells included a pair of orthologous genes (IFI44, IFI44L) that have been reported as interferon stimulated genes (ISGs) that encode microtubule associated proteins with inhibitory activity against hepatitis C virus (HCV) when over-expressed prior to viral infection [29,30]. However, no similar changes were noted in any of 41 other ISGs with antiviral activity [31]. Other notable up-regulated genes in Raji cells included *CCL4*/*CCL4L1*, *L2* and *CCL3L1*. Again, the coordinate regulation of multiple orthologs is supportive of the biological significance of these observations. Moreover, these changes were also fully validated by qtRT-PCR analyses (Figure 5). The *CCL4* and *CCL3* family genes encode chemokines with a common receptor, CCR5 and normally function to attract regulatory T-cells and are a feature of B-cell activation [32].

We also compared the same gene set in FeLV-B infected Raji cells to determine whether these were generic anti-retroviral responses or virus-specific phenomena. Two representative genes of the innate immunity/chemokine subset (IFI44, CCL4) were similarly regulated by FeLV-B and XMLV. Several other genes from the most altered subset shown in Figure 4B were also validated and analysed in FeLV-infected Raji cells. The qt-RT-PCR fold changes mirrored closely the results of the array (blue bars in Figure 5) However, some of these genes were discordant in FeLV and XMLV infected cells, such as the zinc transporter SLC30A4 [33]. This observation is of potential interest as this gene encodes a zinc transporter, while the entry receptors for FeLV-B and XMLV are PIT1, a phosphate transporter [34] and XPR1, a phosphate exporter [35]. Homeostatic links between zinc and phosphate transport are known in plant cells [36], while bacterial phosphate transporters can acts as co-transporters for zinc [37].

## 4. Discussion

In this study we have shown that there is a substantial risk of acquiring *de novo* infection of human xenografts with XMLV. The risk is strain-dependent, and the presence of the *Bxv1* locus which encodes replication-competent XMLV in BALB/c and other commonly used strains [23] is clearly an avoidable risk. The level of risk is also likely to be cell-type dependent, as cells which are highly permissive to virus replication will be more likely to acquire and sustain virus infection. It has been suggested that the transformed phenotype of the prostate cancer cell line 22Rv1 may even be dependent on XMRV, as knockdown cell lines displayed impaired xenograft potential in mice, with increased necrosis and reduced vessel formation [14]. In contrast, the MCF7 cell line used here is not dependent on XMLV infection for tumor formation, although the possibility that infection increases the rate of tumor formation requires further study. Another factor that might account for the ready acquisition of XMLV and related gamma-retroviruses is low expression of APOBEC3G, which appears to be a common feature of prostate cancer cell lines [38] and the MCF7 cell lines used here. However, it is clear that cell lines expressing APOBEC3 mutator activity can also be infected with MLV, as shown by the presence of signature mutations in the virus detected in the Epstein Barr virus-immortalised lymphoblastoid cell line JY [6].

While we did not observe any evidence of XMLV infection arising from passage of cells in NSG mice, we detected single-round transfer of other MLV sequences from explanted cells to uninfected human cells, suggesting the presence of replication-defective viruses with human cell tropism. Notably, a recent study has highlighted the potential of NSG mice to generate ecotropic MLVs, leading to myeloid leukemias of murine origin as a result of engraftment with human cells that secrete paracrine growth factors [39]. The possibility of direct human cell infection by pseudotypes or recombinants with polytropic or xenotropic MLVs in NSG mice therefore cannot be discounted.

Analysis of single cell clones from XMLV infected cultures revealed a surprisingly high copy number (up to 15), suggesting that envelope-mediated interference may be defective in this context. A similar observation was reported recently in HEK-293T cells infected with XMRV which reached a plateau at around 40 copies per cell after several weeks of infection [40]. This phenomenon is likely to increase the risk of insertional mutagenesis, and suggests a need for caution in assessing reports of general phenotypic effects due to infection which could be due instead to secondary integration-dependent events selected during a long passage history e.g., of 22Rv1 cells [14].

We studied infected cells relatively soon after infection (<2 weeks), allowing sufficient time for phenotypic effects to be expressed, but avoiding extensive passage and the chances of post-integration selection due to insertional mutagenesis. Under these conditions subtle phenotypic changes were observed in infected MCF7 cells, which displayed accelerated wound healing. Array analysis revealed *EGR1* as the most markedly regulated gene, and this observation was validated by quantitative RT-PCR. While it is conceivable that *EGR1* plays a role in the altered growth of infected MCF7 cells, the effects of this gene are highly context-specific [41] and will require functional validation.

One of the most interesting features of infection of Raji cells with XMLV was the up-regulation of the *IFI44* and *IFI44L* genes that have been implicated as interferon-stimulated genes that confer resistance to HCV *in vitro*. The role of these genes is not well understood although it is clear they inhibit HCV when expressed prior to infection, apparently at the level of translation and that they are also responsive to cGAS in the absence of a classical interferon response [30,42]. It will be interesting to discover whether these genes also modulate XMLV infection and how they are induced by viral infection. The induction of a set of chemokine genes (*CCL3*, *CCL4* family) with a common receptor (*CCR5*) is also very intriguing and suggests that the effect of infection of XMLV in Raji cells is immunomodulatory. Attraction of regulatory T-cells by this mechanism [32] could conceivably inhibit rather than promote immunity. However, the failure of Raji cells to form tumours here is suggestive of the latter scenario. A further issue to be considered is the EBV infected status of Raji Burkitt’s lymphoma cells. While these cells display only the minimal ‘latency I’ pattern of EBV with expression of EBNA-1 and non-coding RNAs, they can be induced to express immediate early lytic cycle genes. It would be interesting to test whether non-EBV infected Burkitt’s lymphoma cell lines display a similar response to gammaretroviral infection, and whether EBV functions are affected in Raji cells [43]. Whether EBV-related or not, it will be of considerable interest to discover the generality of this phenomenon and the mechanism by which XMLV induces these chemokine genes. The idea that this innate immune response by transformed leukemia cells could be replicated safely and harnessed to assist immune recognition and clearance of tumor cells is attractive and merits future consideration.

## Supplementary Information

Figure S1: Alignment of *de novo* rescued XMLV from MCF7-BALB/c xenografts with the *Bxv1* locus.

Figure S2: Accelerated scrape wound healing in XMLV infected compared to uninfected MCF7 cells.

Supplementary data 1: ImageJ analysis of scrape wound assay in MCF7 cells

Supplementary data 2: Gene Expression changes in XMLV-infected MCF7 and Raji cells (Excel file).
